# Special theme article: science and sociology of footwear

**DOI:** 10.1186/s13047-018-0293-y

**Published:** 2018-09-14

**Authors:** Anita Ellen Williams

**Affiliations:** Directorate of Prosthetics, Orthotics and Podiatry, Univeristy of Salford, Salford, UK

The design and construction of footwear is constantly evolving in order to maximise its role of providing protection for the external environment, accommodate bony and soft tissue problems and assist in the biomechanical function of the foot, ankle and lower limb. The drive for this is evident in the papers in this issue that demonstrate how footwear contributes to foot pathology [1], pain [2], ulcers and falls [3]. Hence, we are pleased to present in the Journal of Foot and Ankle Research, a broad range topics related to footwear that are highly relevant to clinical practice, the manufacturers of footwear and to future research directions. The call for papers for this themed issue has resulted in 5 new articles and 8 published articles which cover the wide ranging aspects of footwear.

Appropriate footwear is vital to maximising the potential for good foot health throughout a person’s life. However, footwear choices are dependent on many influencing factors such as underlying pathology/symptoms, gender, work and social demands to name a few [4]. As such, this issue emphasises the complexities associated with the interrelationship between footwear, feet and the person wearing it. This themed issue provides the reader with information on footwear problems in people with inflammatory arthritis [5], lupus [6] diabetes [7] and neuromuscular conditions that include stroke and Parkinson [8]. The direct effect of pathological process and the resultant changes in the feet mean that people with long term foot conditions experience high levels of foot pain, impairment and disability. These problems can be compounded by the use of inappropriate footwear. Indeed, this issue emphasises that people with long term foot conditions frequently wear footwear which is ill-fitting and this is either due to it being an inappropriate design at the point of purchase or that the use over time has resulted in effective design components becoming ineffective [﻿3﻿]. Our increased understanding of factors related to footwear in different populations and in different contexts have provided the basis for how these problems may be solved through the evolution of footwear design and how patients are supported with information on appropriate footwear for various activities including work.

The footwear worn in the workplace is often not the footwear that we see in the clinical setting and hence insight into the impact of footwear worn in the work environment may aid diagnosis of foot and lower limb problems and inform management plans. Hence, footwear issues of fit and comfort in workers who stand for long periods of time are covered in this issue [9]. Slips, trips and falls in an occupational setting are considered to be key incidents that account for lower limb injury and consequently lost time and days lost. The identification of occupational risk factors and the solutions are multi-faceted with footwear being a key component in these incidents.

Paediatric footwear is an aspect that is under investigation in relation to the perceived and potential effects on the development of the foot over time [10]. The emerging findings from the same research team [11] support the need for progress in children’s footwear science and advance understanding of the interaction between the foot and shoe in order to improve our care and understanding of children’s feet.

Also presented for the readership are advances in our understanding of the biomechanical considerations of footwear related to foot motion [12]. These insights into foot and ankle biomechanics will become even more essential as we investigate and treat complex diseases. Further, in relation to footwear design, research demonstrates that particular design features are beneficial in managing specific conditions, for example rockers soles for people with diabetes [13] and neoprene uppers reduce discomfort in the toe area [14].

However, not only is it important to ensure the design of footwear is correct but that people are supported in choosing the most appropriate footwear. Guidelines can inform practitioners on what needs to be considered [15] and clinical tools aid the level of understanding that the clinician needs in order to support the patient in their footwear choices [4].

In conclusion, the intention of this special theme issue is to spark intellectual curiosity, and generate interest in the importance of footwear, which will lead to new insights, better understanding and ultimately improved foot health for people throughout their lives.

Anita Williams PhD

Associate Editor, Journal of Foot and Ankle Research
